# Untreated anti-Ca/ARHGAP26 autoantibody-associated cerebellar ataxia progressing over 27 years

**DOI:** 10.1007/s00415-023-11632-2

**Published:** 2023-03-01

**Authors:** E. Schegk, I. Beiser, L. Achtnichts, K. Nedeltchev, M. Bertschi, M. Gschwind

**Affiliations:** 1 Department of Neurology, Cantonal Hospital Aarau, Tellstrasse 25, CH-5011 Aarau, Switzerland; 2 Department of Neurology, University Hospital and University of Berne, Berne, Switzerland; 3 Department of Neurology, Geneva University Hospitals and University of Geneva, Geneva, Switzerland

Dear Sirs,

Autoantibodies against the RhoGTPase-activating protein 26 (ARHGAP26) and autoantibody-associated cerebellar ataxia were first described in 2010 [1]. We present a case of anti-Ca-associated cerebellar ataxia with Parkinson-like traits and a so far never described very slow progression over 27 years without treatment, therefore, broadening clinical presentation by parkinsonian features.

A 79-year-old farmer presented to our department with a movement disorder that had been slowly progressing over a time span of 27 years.

His neurologic history started in 1992, when the so far healthy patient in his mid-50 s received neurologic work-up for the first time because of involuntary movements that had started 2 years before (first in the right arm, then leg, and after 6 months also in left arm and leg). He then further complained about myoclonic jerks triggered by targeted movements and a freezing gait with consecutive falls, he also described an incapacity of unclasping objects, which led to broken cups. Laboratory testing showed normal findings, notably normal ceruloplasmin levels, and a normal infectiology work-up. Oligoclonal bands in the cerebrospinal fluid (CSF) were positive. Electroencephalography and motor evoked potentials were normal. No autonomic neuropathy was found. Brain MRI displayed frontoparietal cortical atrophy, MRI of the cervical spine was normal. Single-photon emission computed tomography (SPECT) and positron emission tomography (PET) of the brain were normal. Consequently, our patient had been diagnosed with multiple system atrophy of the olivo-ponto-cerebellar subtype (MSA OPC), and treatment with selegiline and levodopa/benserazide was established.

In 1996, freezing of gait and falls had improved under dopaminergic treatment, whereas the myoclonic jerks had worsened. He furthermore suffered from a repeated loss of consciousness under orthostatic conditions. Clinical examination revealed slight stammering, a positive palmomental reflex more pronounced right than left and myoclonic jerks when the right limbs were passively flexed. He showed intention myoclonus in the finger-nose and heel-shin test. A rebound was seen in the upper extremities. No signs of cognitive decline or apraxia were documented. The brain MRI confirmed atrophy of both cerebrum and cerebellum. Levodopa/benserazide was continued, Selegiline was tapered.

During the following 20 years after this initial evaluation, no neurologic follow-up would take place until the patient was admitted to our department because of an acute worsening of his myoclonic jerks. He now reported that the previously documented gait freezing with postural imbalance was accompanied by morning stiffness, hypomimia, hypophonia, and dysarthria, cognitive decline with forgetfulness and involuntary loss of urine. Clinical examination showed a slowed and depressed patient, with saccadic eye movements, hypometric saccades, rigidity of the left arm and both legs, resting tremor of all limbs, predominant at the left hand, irregular posture tremor of the hands, with re-emerging as well as stimulus sensitive myoclonic jerks of all limbs and apraxia. Laboratory testing showed a urinary tract infection, it was treated with Ceftriaxone 2 g/d for 2 days.

The debilitating jerks improved partially under clonazepam 0.25 mg/d. Levodopa test showed only minimal improvement from 42 to 39 points (MDS-UPDRS III), mainly in the upper extremities, whereas the marked axial symptoms were unchanged. Electrophysiological testing was normal. A new brain MRI revealed frontoparietal cortical atrophy but no atrophy of the basal ganglia or the cerebellum and no signs of normal pressure hydrocephalus (Fig. 1). Neuropsychologic testing revealed an unspecific, mild cognitive impairment (verbal learning weakness).

**Fig. 1:**
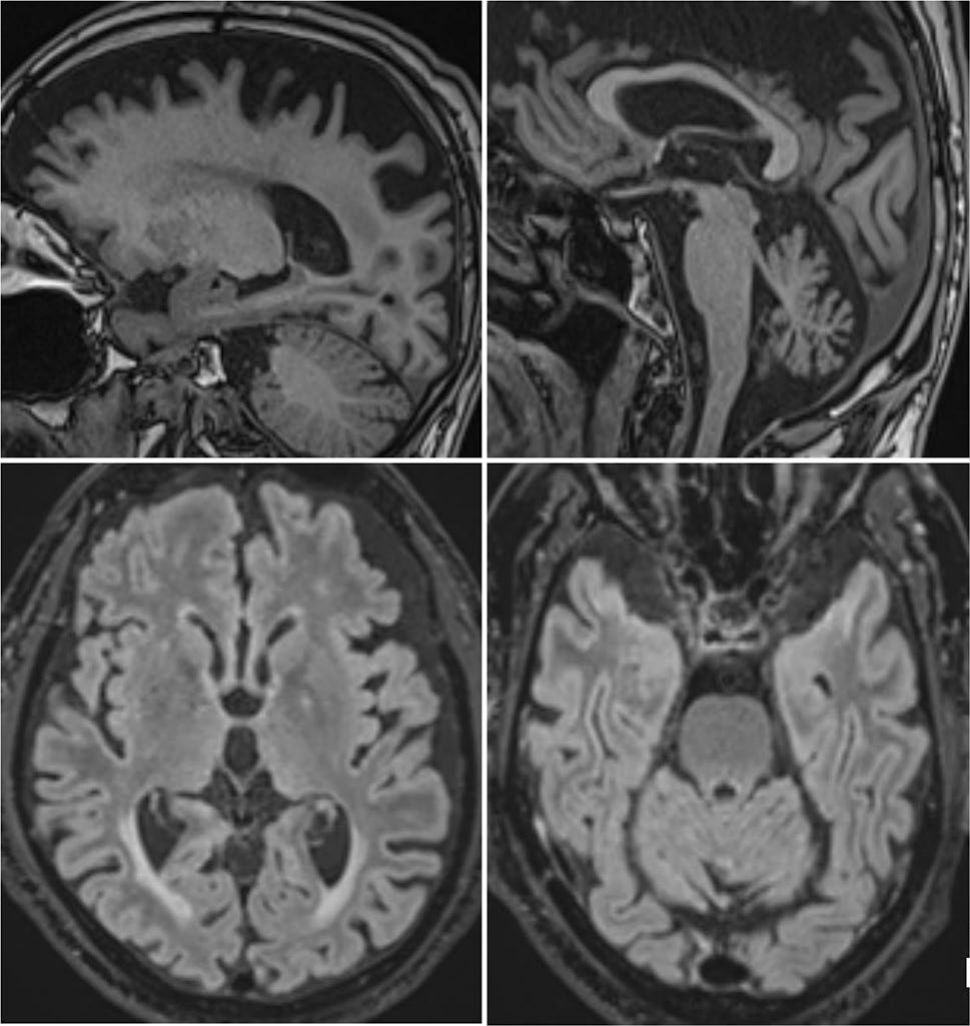
New brain MRI of the patient. Sagittal MRI-images (MPRAGE) and axial FLAIR-weighted images of our patient showing frontoparietal cortical atrophy and cerebellar, vermis-accentuated atrophy

CSF was unchanged with a normal cell count, but positive oligoclonal bands. For that reason, we also searched for an autoimmune or paraneoplastic process. The panel of anti-neuronal autoantibodies (anti-Hu, -Ri, -Yo, -amphiphysin, -CV2, -Ma, -Ta/Ma2, -Sox1, -Tr, -Zic4, -NMDA-R, -VGCC, -VGKC, -LGI1, -CASPR2, -GAD) was negative, as were rheumatic antibodies (ANA, ANCA, rheumatoid factor). A whole-body FDG-PET/CT was normal, giving no evidence for a neoplastic process.

Results from cell-based indirect immunofluorescence on monkey cerebellum arrived after the patient had been discharged. Rho GTPase-activating protein 26 had been detected (Titer 1:1000, considered highly significant), consistent with autoantibody-associated cerebellar ataxia. Unfortunately, the patient later refused further follow-up.

This patient, diagnosed 27 years ago with a multisystem atrophy of the olivo-ponto-cerebellar subtype in 1993, and found to have ARHGAP26 autoantibody-associated cerebellar ataxia today, showed a very slow evolution. Interestingly, the parkinsonian motor symptoms were predominant with freezing, falls, and fluctuating myoclonic jerks. There was autonomic dysregulation but there were no pyramidal signs. Anti-ARHGAP26 antibodies bind to all structures of Purkinje cells. They are also detectable in a subset of neurons in the hippocampus [2]. Involvement of the basal ganglia, which could have explained the extrapyramidal signs of our patient, was, to our knowledge, never described.

From today’s retrospective perspective, the patient’s symptomatology and neuroimaging is not completely consistent with the diagnosis of olivo-ponto-cerebellar atrophy. Especially, in the initial description, there was no ataxia or cerebellar dysarthria described; instead, involuntary jerks and signs of dysautonomia are present. The cerebellar syndrome would seem to correspond to a cognitive affective syndrome, but this syndrome is not specific for cerebellar damage in the absence of clear neurological signs that would depose cerebellar damage. Only frontoparietal but not cerebellar atrophy is reported in the first MRI, and SPECT and PET scans were judged unremarkable. In the 1996, MRI both frontoparietal atrophy and cerebellar atrophy were found, however, other markers of OPCA such as atrophy of middle cerebellar peduncles were not. We underline that we referred the historic medical documentation of that time. From today’s perspective, we might, therefore, better describe our patient as an unusual case of movement disorder with the presence of anti-ARHGAP26 autoantibody-associated cerebellar ataxia, in which, however, olivo-ponto-cerebellar atrophy may be one of the diagnostic hypotheses considered.

So far, a total of ten cases of anti-ARHGAP26 autoantibody-associated cerebellar ataxia have been published, five among them of possible paraneoplastic origin [3–6]. The disease mostly manifests relatively subacute within a few days [1, 3, 4]. However, in some cases, symptoms included dizziness, ataxia, dysarthria [6], behavioral changes [7], and cognitive decline [5] over several months to years until the diagnosis could be made. The long-term course of the disease is largely unknown. In one patient, diagnosed after 18 years of disease onset [3], treatment with valproate (because of an initially suspected myoclonic epilepsy) resulted in a 5-year remission. Another patient showed limited benefit after immunosuppressive therapy right after disease onset, but no benefit from escalated immune therapy at a later stage [4], contrasting to a third patient who reported transient benefit from intravenous methylprednisolone 19 years after disease onset [5]. The rate of progression may, therefore, be a source of heterogeneity for this autoantibody.

Patients with cerebellar dysfunction may struggle with depression, limitations in cognitive ability and slowed reaction time, known as the cerebellar cognitive affective syndrome [2, 6–8], which was also found in our patient. He also displayed deficits in verbal learning and recall, which was previously described in anti-ARHGAP26 antibody-positive patients [4, 6] and is consistent with limbic system involvement. ARHGAP26 is expressed in a subset of neurons in the hippocampus, which could explain those symptoms.

Our case illustrates that anti-ARHGAP26 antibody-positive patients can present a very slow subacute evolution. However, to date, we cannot exclude that the anti-ARHGAP26 antibody was in fact an accidental finding and not causally related to the patient’s condition. During the last 20 years, major advances in knowledge about autoimmune and paraneoplastic cerebellar ataxias have been made, and many new antibodies have been characterized [9]. The additional clinical description of our patient is, therefore, highly valuable, all the more as he was diagnosed by an expert in the field 27 years ago.

## Supplementary Information

Below is the link to the electronic supplementary material.

Video: Our patient at hospital admission with severe ataxia and two days later, after treatment initiation. Supplementary file1 (MP4 161844 KB)

## Data Availability

No consent was given for public data sharing. Data are available from the corresponding author on reasonable request.
